# A national estimate of the birth prevalence of congenital anomalies in India: systematic review and meta-analysis

**DOI:** 10.1186/s12887-018-1149-0

**Published:** 2018-05-25

**Authors:** Prajkta Bhide, Anita Kar

**Affiliations:** Interdisciplinary School of Health Sciences, Savitribai Phule Pune University, Pune, 411007 India

**Keywords:** Birth defects, Congenital anomalies, Congenital malformations, Birth prevalence, India, Meta-analysis

## Abstract

**Background:**

A quarter of all global neonatal deaths occur in India. Congenital anomalies constitute the fifth largest cause of neonatal mortality in the country, but national estimates of the prevalence of these conditions are lacking. The objective of the study was to derive an estimate of the birth prevalence of congenital anomalies in India.

**Methods:**

The search was carried out in PubMed and pooled prevalence was estimated using the inverse variance method. A random effects model was used due to high heterogeneity between the studies. Forest plots were generated using the Review Manager software.

**Results:**

The PubMed search identified 878 articles from which 52 hospital based and three community based studies were included in the meta-analysis. The pooled prevalence of congenital anomaly affected births was 184.48 per 10,000 births (95% CI 164.74–204.21) among 802,658 births. Anomalies of the musculoskeletal system were highest among live births while the prevalence of central nervous system defects was highest when stillbirths were included in the analysis. Anencephaly and talipes were the most commonly reported anomalies.

**Conclusions:**

Data from this meta-analysis suggests that there may be as many as 472,177 (421,652 to 522,676) congenital anomaly affected births in India each year. Population based studies using standard definitions are needed to validate these estimates. The two most frequently reported anomalies were anencephaly that is potentially preventable through preconception folate supplementation, and talipes which can be corrected using relatively low cost interventions. Studies are needed to determine the impact of congenital anomalies on neonatal mortality in India.

## Background

A quarter of global neonatal deaths occur in India. In 2013, the country reported a neonatal mortality rate of 29 per 1000 live births, responsible for 753,000 neonatal deaths [1]. While the highest contributors to neonatal deaths were preterm births (34.7%), intrapartum complications (19.6%), pneumonia (16.3%) and neonatal sepsis (15%), congenital anomalies constituted the fifth largest cause, being responsible for an estimated 9% of neonatal deaths in the year 2010 [2]. There is evidence of transition in causes of infant and child mortality in low and middle-income countries, including India [3]. With a decrease in infectious causes of infant deaths, especially in urban areas in India, the proportion of mortality due to congenital anomalies is likely to increase [4]. Global estimates suggest that congenital anomalies affect 2–3% of births [5]. Assuming a 2% birth prevalence, and 25,595,000 births in 2013 [6], an estimated 511,900 births may have been affected with a congenital anomaly in India. These estimates exceed the combined totals of anomaly affected births occurring in several high-income countries [7]. The true magnitude of the number of births affected by congenital anomalies in India is unknown due to lack of a national birth defects surveillance. The need for data arises as currently there is no data on the impact of congenital anomaly affected pregnancies or births on health service utilization, for either termination of pregnancy due to detection of a fetal anomaly or for neonatal intensive care services. Another requirement for data is to derive estimates of the number of children born with disabling conditions. Medical and rehabilitative services for affected children through government health services are currently limited in India, resulting in significant out of pocket expenditure for families [8, 9]. Data on the magnitude of congenital anomalies are also needed as some of these conditions can be prevented through primary care interventions targeted towards women in the preconception, intra-conception and antenatal periods [10]. Strategies targeting the prevention of births affected by congenital anomalies also target the shared risk factors for other adverse pregnancy outcomes, effectively aiming at reduction of reproductive wastage, and improving pregnancy outcome [11]. In this study, we systematically reviewed available Indian studies, in order to derive a national estimate of births affected by congenital anomalies in India. We also discuss the implications of this quantitative analysis in terms of prevention and care, further research needs, and the characteristics of a birth defects surveillance system in India.

## Methods

### Search strategy

A literature search was performed in PubMed in April 2015 using the keywords: (“congenital abnormalities”[MeSH Terms] OR (“congenital”[All Fields] AND “abnormalities”[All Fields]) OR “congenital abnormalities”[All Fields] OR (“congenital”[All Fields] AND “anomalies”[All Fields]) OR “congenital anomalies”[All Fields]) AND (“epidemiology”[Subheading] OR “epidemiology”[All Fields] OR “prevalence”[All Fields] OR “prevalence”[MeSH Terms]) AND (“india”[MeSH Terms] OR “india”[All Fields]). No restrictions were used for date of publication. Further searches were carried out among the reference lists of eligible articles.

### Study selection

All titles and abstracts identified in the PubMed search were screened for the possibility of extracting birth prevalence data. Studies were eligible to be included in the review if they fulfilled the following inclusion criteria: 1) reported data on the number of anomaly affected babies or anomalies identified at birth among either live born and/or stillborn babies and 2) were conducted in India. Exclusion criteria: 1) Case reports and papers focusing on etiology, diagnosis or clinical management were excluded. 2) Studies that reported prevalence data of only a single anomaly or system were not included in the analysis as these represented non-random, selected cases, and would therefore distort prevalence estimates.

### Data quality

Studies were included if: 1) a clear description of study setting (hospital or community-based) was mentioned, 2) study reported total number of births in the given time period, and 3) number and type (live or stillbirth) of anomaly affected births amongst the total births was mentioned.

### Data extraction

A data extraction form was designed in MS Excel for the following study characteristics: study and geographical setting, study duration, sample size, and primary outcomes of interest which included the number of anomaly affected babies or the number of anomalies and the number of births (live and stillbirths) as reported in the study. When a study was eligible for inclusion in the review, the numerator and denominator were verified and the prevalence estimate was recalculated.

### Statistical analysis

Birth prevalence of congenital anomalies was calculated as the total number of babies (both live born and stillborn) with anomalies per 10,000 births [12]. The live birth prevalence was determined from the number of anomaly affected live births per 10,000 live births [12]. Pooled prevalence was estimated in Review Manager (version 5.3) software using the inverse variance method. Due to the high heterogeneity between studies (*I*^2^ > 95%, *p* < 0.05) the meta-analysis was conducted using a random-effects model.

## Results

### Search results

**Fig. 1:**
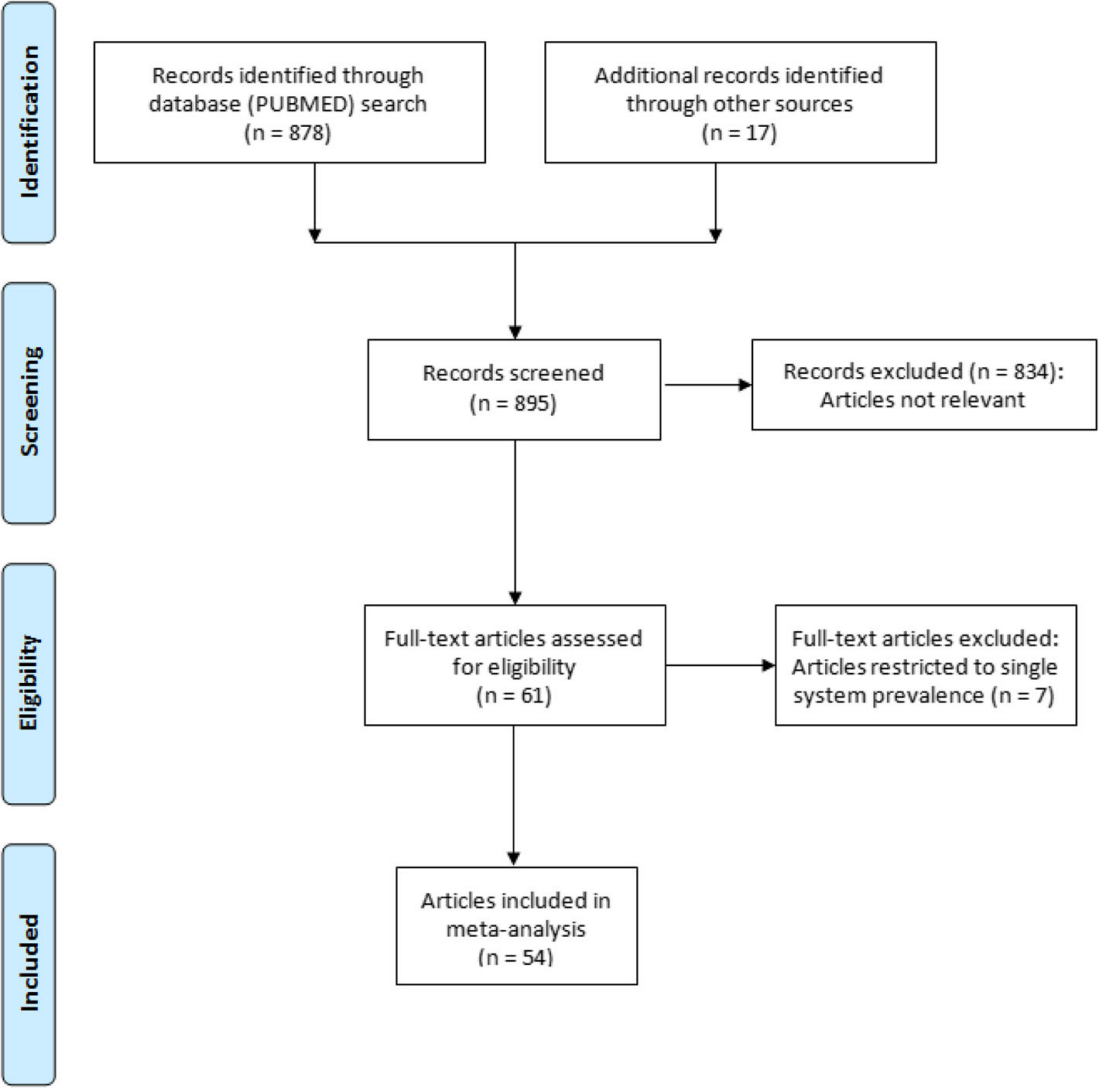
Search strategy and selection of studies: PRISMA flowchart

The PubMed search identified 878 articles, of which only 50 articles were identified to be of potential interest for inclusion in the review. A search of the reference lists of these 50 articles yielded a further 17 articles of potential interest, published in non-indexed Indian journals. Finally 54 articles fulfilled the inclusion criteria and were included in the review (Fig. 1).

### Study characteristics

There were 52 hospital-based [13–63] and three community-based studies [64–66] (one article [58] reported two separate studies conducted in Mumbai (39,498 births) and Kolkata (19,191 births)). These 55 studies were reported between 1960 and 2015 (Table 1). Clinical examination was the major method of case ascertainment and was backed up only in 14 studies by radiological, ultrasound and other investigations [17, 20, 22, 23, 26–28, 33, 40, 48, 50–52, 60]. Nine studies reported the involvement of neonatologists or pediatricians in case ascertainment [19, 26, 35, 39, 45, 49, 51, 62, 64]. The autopsy rate varied among the hospital studies ranging from 0 to 25% for stillbirths and early neonatal deaths. None of the studies reported the number of pregnancies terminated due to detection of fetal anomalies. Community studies were restricted to live births and did not report stillbirths and early neonatal deaths.

**Table 1:** Characteristics of studies included in the review (*n* = 55)

Study	Study period	Study setting	Duration	Place	Number of births	Number of anomaly affected births	Birth prevalence per 10,000 births
Agarwal et al. 1991 [13]	1981–1984	hospital	31.5 months	Lucknow	9405 births	192	204.15
Agarwal et al. 2014 [14]	2010–2011	hospital	12 months	Bhubaneswar	7268 births	116	159.6
Aiyar and Agarwal 1969 [15]	1966–1967	hospital	19 months	Mumbai	10,000 live births	172	172^a^
Anand et al. 1988 [16]	NM	hospital	NM	Jamnagar	2000 births	40	200
Bai et al. 1982 [17]	NM	hospital^b^	12 months	Trivandrum	7167 births	132	184.18
Bai et al. 1990 [18]	NM	hospital	12 months	Trivandrum	13,964 births	50	35.81
Baruah et al. 2015 [19]	2010–2013	hospital	34 months	Dibrugarh	18,192 births	206	113.24
Bharucha 1998 [20]	1993–1996	hospital^b^	39 months	Mumbai	42,304 births	972	229.77
Bhat and Babu 1998 [21]	1989–1992	hospital	40 months	Pondicherry	12,797 births	353	275.85
Chaturvedi and Banerjee 1989 [22]	1985–1986	hospital^b^	12 months	Wardha	3014 births	82	272.06
Chinara and Singh 1982 [23]	1978–1979	hospital^b^	12 months	Varanasi	1774 births	37	208.57
Choudhary et al. 1984 [24]	1976–1980	hospital	60 months	Kolkata	21,016 births	63	29.98
Choudhary et al. 1989 [25]	1976–1987	hospital	120 months	Kolkata	126,266 births	535	42.37
Christopher and Jadhav 1986 [26]	1979–1983	hospital^b^	60 months	Vellore	21,585 births	131	60.69
Desai and Desai 2006 [27]	NM	hospital^b^	12 months	Mumbai	2188 births	79	361.06
Dutta and Chaturvedi 2000 [28]	1998–1999	hospital^b^	13 months	Wardha	2968 births	37	124.66
Duttachoudhary and Pal 1997 [29]	1991–1993	hospital	36 months	Durgapur	7242 births	26	35.9
Ghosh et al. 1979 [30]	1974–1976	hospital	29 months	Kolkata	2019 births	29	143.64
Ghosh et al. 1985 [64]	1969–1973	community	40 months	New Delhi	7590 live births	189	249.01^a^
Goravalingappa and Nashi 1979 [31]	1986–1987	hospital	15 months	Hubli	2398 births	75	312.76
Grover 2000 [32]	1991–1995	hospital	60 months	Shimla	10,100 births	180	178.22
Hemrajani et al. 1971 [33]	1965–1969	hospital^b^	60 months	Jaipur	28,511 births	608	213.25
Jaikrishan et al. 1999 [34]	1995–1998	hospital	41 months	Kerala	36,805 births	538	146.18
Jaikrishan et al. 2013 [35]	1995–2011	hospital	191 months	Kerala	141,540 births	1370	96.79
Joseph et al. 2010 [65]	2004–2005	community	6 months	Belgaum	194 live births	4	206.19^a^
Khanna and Prasad 1967 [36]	1964	hospital	9 months	Patna	5376 births	74	137.65
Kolah et al. 1967 [37]	1960–1963	hospital	39 months	Mumbai	23,568 births	331	140.44
Kulkarni et al. 1987 [38]	1984	hospital	6 months	Davangere	2000 births	81	405
Kulshetra et al. 1983 [66]	1976–1977	community	24 months	Ballabhgarh	2409 live births	79	327.94^a^
Marwah et al. 2014 [39]	2010–2011	hospital	12 months	Patiala	1554 births	69	444.02
Mathur et al. 1975 [40]	1970	hospital^b^	4 months	Hyderabad	1060 births	33	311.32
Mishra and Baveja 1989 [41]	1983–1987	hospital	48 months	Allahabad	4098 births	60	146.41
Mital and Grewal 1969 [42]	1967–1968	hospital	15 months	Kanpur	4150 births	93	224.1
Modi et al. 1998 [43]	1993–1997	hospital	40 months	Baroda	31,775 births	651	204.88
Parmar et al. 2010 [44]	2006–2007	hospital	18 months	Bhavngar	4210 births	37	87.89
Patel and Adhia 2005 [45]	NM	hospital	24 months	Mumbai	17,653 births	294	166.54
Patel et al. 2014 [46]	2012–2014	hospital	24 months	Ahmedabad	16,481 births	210	127.42
Rao et al. 2014 [47]	2008–2012	hospital	60 months	Mangalore	28,373 births	344	121.24
Ronya et al. 2002 [48]	1999–2000	hospital^b^	9 months	Wardha	3000 births	62	206.67
Sachadeva et al. 2014 [49]	2010	hospital	4 months	Rohtak	2862 live births	47	164.22^a^
Saifulla et al. 1967 [50]	1966	hospital^b^	8 months	Chandigarh	1000 births	36	360
Sarkar et al. 2013 [51]	2011–2012	hospital^b^	12 months	Kolkata	12,896 live births	286	221.77^a^
Savaskar et al. 2014 [52]	2011–2013	hospital^b^	NM	Latur	10,294 births	443	430.35
Shah and Pensi 2013 [53]	NM	hospital	9 months	Ahmedabad	4456 births	106	237.88
Sharma 1970 [54]	1967–1969	hospital	36 months	Mysore	5554 births	14	25.21
Sharma et al. 1972 [55]	1963–1964	hospital	14 months	Lucknow	2851 births	40	140.3
Singh and Gupta 2009 [56]	2002	hospital	12 months	Jammu	9308 births	140	150.41
Singh and Sharma 1980 [57]	1975–1978	hospital	48 months	New Delhi	6274 live births	170	270.96^a^
Stevenson et al. 1966 [58]	1961–1964	hospital	NM	Mumbai (Purandare VN)	39,498 births	340	86.08
Stevenson et al. 1966 [58]	1961–1964	hospital	NM	Kolkata (Mitra KN)	19,191 births	59	30.74
Swain et al. 1994 [59]	1988–1989	hospital	24 months	Varanasi	3932 births	48	122.08
Taksande et al. 2010 [60]	2005–2007	hospital^b^	31 months	Wardha	9386 births	179	190.71
Tibrewala and Pai 1974 [61]	1968–1972	hospital	60 months	Mumbai	12,360 live births	232	187.7^a^
Verma et al. 1991 [62]	1983–1989	hospital	75 months	Ludhiana	10,000 births	359	359
Verma et al. 1998 [63]	NM	hospital	36 months	New Delhi	23,367 births	433	185.3

*NM* not mentioned

^a^Studies reporting live births only. Prevalence has been reported per 10,000 live births. All other studies reported both live and stillbirths

^b^Apart from these hospital studies which used radiological, ultrasound and some other investigations, case ascertainment was done only through physical assessment

### Birth prevalence of congenital anomalies

**Fig. 2:**
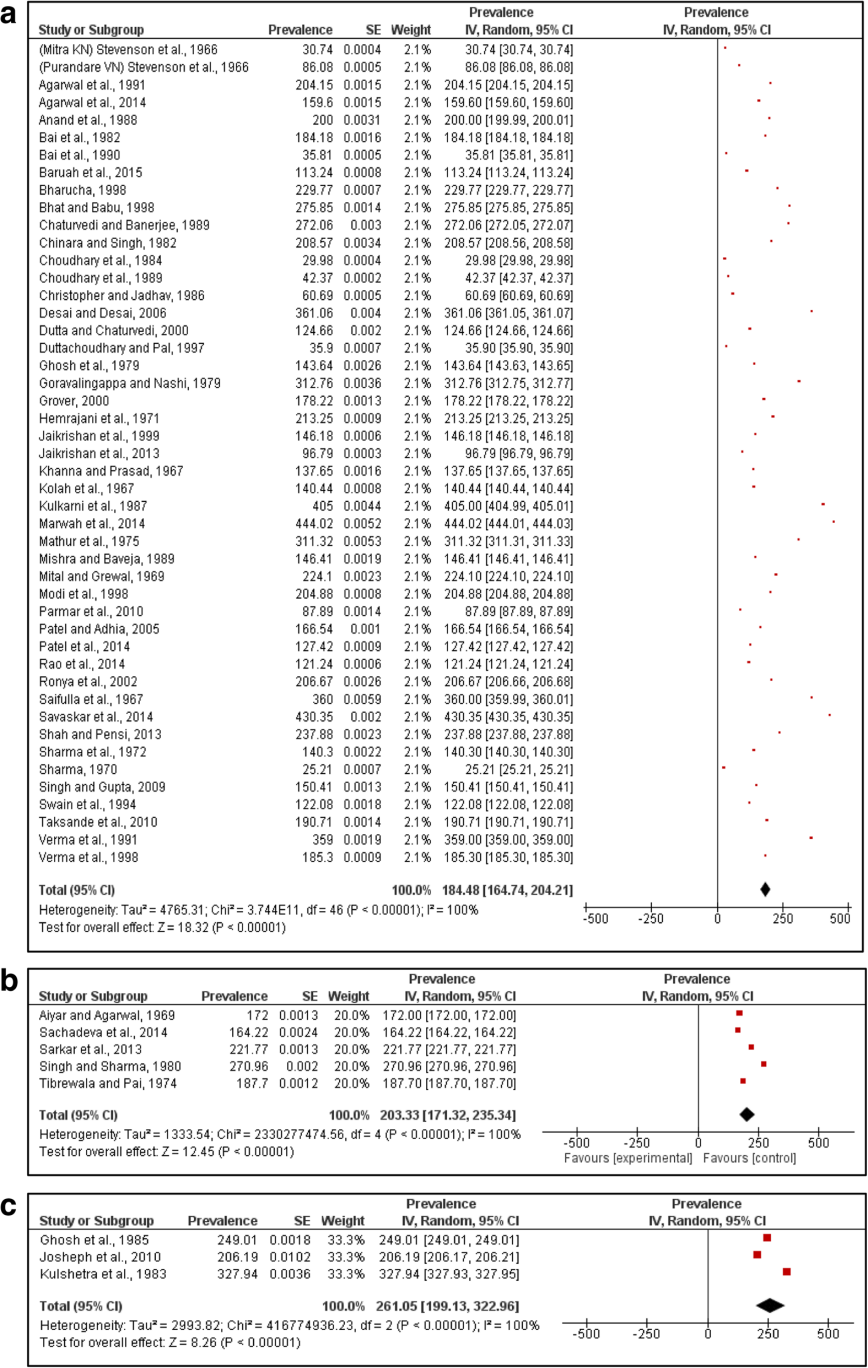
Pooled prevalence of congenital anomalies. **a.** pooled prevalence of congenital anomaly affected births (both live and stillbirths) in hospital setting. **b.** pooled prevalence of congenital anomaly affected live births (hospital setting). **c.** pooled prevalence of congenital anomaly affected live births (community setting)

Data on births affected by congenital anomalies was reported from 52 hospital-based studies of which 47 studies reported both live and stillbirths while five studies reported only live births. The number of births screened ranged from 1000 to 141,540 for hospital-based studies. The reporting of anomalies was done during the period of hospital stay till discharge. The pooled prevalence of congenital anomaly affected births from 802,658 births using a random-effects model was 184.48 per 10,000 births (95% CI 164.74–204.21) (Fig. 2a). The five hospital-based studies reported a pooled live birth prevalence of 203.33 per 10,000 live births (95% CI 171.32–235.34) for 44,392 live births (Fig. 2b).

Community-based studies reported the prevalence of anomaly affected births within the first week post birth. The studies reported screening of live births ranging from 194 to 7590 live births. The pooled prevalence for community studies from 10,193 live births was 261.05 per 10,000 live births (95% CI 199.13–322.96) (Fig. 2c). Due to paucity of studies, analysis of congenital anomaly prevalence rates over time did not yield meaningful results.

### System-wise prevalence of anomalies

Table 2 presents the system-wise prevalence of anomalies. Among hospital studies, which included data on both live births and stillbirths, anomalies of the central nervous system were most frequently reported, followed by anomalies of the musculoskeletal system (75.85 per 10,000 births (95% CI 58.80–92.90) and 65.64 per 10,000 births (95% CI 52.97–78.31), respectively). Cardiovascular system anomalies had the lowest birth prevalence across both hospital and community settings. Among live births, anomalies of the musculoskeletal system were highest in both hospital (79.38 per 10,000 live births (95% CI 32.32–126.44)) and community settings (65.88 per 10,000 live births (95% CI 23.13–108.63)). The corresponding prevalence of central nervous system defects was lower (28.93 per 10,000 live births (95% CI 13.64–44.22) for hospital-based studies and (26.19 per 10,000 live births (95% CI 15.55–36.83) for community-based studies).

**Table 2:** Pooled prevalence of anomalies by affected systems

System	Birth prevalence per 10,000 births (*n* = 14 hospital studies)	Live birth prevalence per 10,000 live births (*n* = 3 hospital studies)	Live birth prevalence per 10,000 live births (n = 3 community studies)
Central nervous system	75.85 (95% CI 58.80–92.90)	28.93 (95% CI 13.64–44.22)	26.19 (95% CI 15.55–36.83)
Musculoskeletal system	65.64 (95% CI 52.97–78.31)	79.38 (95% CI 32.32–126.44)	65.88 (95% CI 23.13–108.63)
Cardiovascular system	27.06 (95% CI 20.03–34.09)	23.04 (95% CI 4.69–41.39)	9.32 (95% CI -0.81 - 19.45)
Gastrointestinal system	50.19 (95% CI 42.50–57.87)	37.72 (95% CI 26.41–49.03)	a
Genitourinary system	39.08 (95% CI 27.86–50.30)	28.41 (95% CI 16.18–40.65)	37.42 (95% CI 13.14–61.70)

^a^Data not analyzed due to misclassification of umbilical hernia as gastrointestinal system anomalies

### Prevalence of selected anomalies

Table 3 presents pooled prevalence of certain frequently reported congenital anomalies among hospital studies. Anencephaly was the most commonly reported anomaly with a birth prevalence of 21.1 per 10,000 births (95% CI 16.91–25.29) followed by talipes (birth prevalence 17.9 per 10,000 births (95% CI 15.09–20.71)), orofacial clefts (birth prevalence 14.94 per 10,000 births (95% CI 12.64–17.24)) and hypospadias (birth prevalence 12.20 per 10,000 births (95% CI 9.79–14.60)) among the 25 studies examining all births occurring in the hospital. Among hospital studies that excluded stillbirth data, the pooled prevalence of talipes (35.08 per 10,000 live births, 95% CI 16.88–53.29) was higher than anencephaly (17.11 per 10,000 live births, 95% CI 13.59–20.63) among live births.

**Table 3:** Pooled prevalence of selected anomalies

Anomaly	Birth prevalence per 10,000 births (*n* = 25 hospital studies)	Live birth prevalence per 10,000 live births (*n* = 5 hospital studies)
Anencephaly	21.10 (95% CI 16.91–25.29)	17.11 (95% CI 13.59–20.63)
Exomphalos / omphalocele	4.65 (95% CI 3.23–6.07)	1.60 (95% CI 0.46–2.74)
Gastrochisis	7.00 (95% CI 4.56–18.56)	1.60 (95% CI 1.60–1.60)
Hypospadias	12.20 (95% CI 9.79–14.60)	5.39 (95% CI 3.19–7.59)
Orofacial clefts	14.94 (95% CI 12.64–17.24)	15.69 (95% CI 11.74–19.63)
Spina bifida	5.85 (95% CI 4.48–7.21)	8.45 (95% CI 3.08–13.81)
Talipes	17.90 (95% CI 15.09–20.71)	35.08 (95% CI 16.88–53.29)

## Discussion

Congenital anomalies are not prioritized as public health problems in low income countries as they are considered to be rare conditions that are self-limiting due to the high mortality of affected infants [67]. Another reason for under-prioritization of these conditions is the understanding that most birth defects are not preventable through low-cost primary care strategies, the major approach of public health services of low income countries. In this study, we derived a national estimate of the birth prevalence of congenital anomalies occurring in India, as such data are currently unavailable due to lack of birth defects surveillance. Using a systematic literature search followed by meta-analysis, we derived a pooled prevalence of congenital anomaly affected births of 184.48 per 10,000 births (95% CI 164.74–204.21). This prevalence is slightly lower than that reported by the European Surveillance of Congenital Anomalies registry (215.54 affected births per 10,000 births (95% CI 214.14–216.94)) [68]. In terms of absolute numbers, however, these estimates indicate that congenital anomalies are not rare events in India, as the data suggests that between 421,652 to 522,676 anomaly affected births may be occurring in the country each year. Due to the reporting of stillbirths in hospital-based studies, anencephaly was the most frequently reported anomaly, followed by talipes, orofacial clefts and hypospadias. Neural tube defects (NTDs) like anencephaly are potentially preventable through a low cost primary prevention method of preconception folic acid supplementation [69, 70], but there are as yet no national guidelines on folic acid fortification/supplementation in India. Combined with preconception iron supplementation, this primary care intervention could not only reduce the number of NTDs in the country, but also reduce anemia, a persistent maternal health challenge in low income countries [71]. Community-based studies reported a higher prevalence of musculoskeletal anomalies, with talipes, a potentially treatable anomaly, being reported as the most common congenital anomaly among live births. Thus, in addition to determining the large numbers of affected births, this review identified that the two most commonly reported congenital anomalies were preventable/treatable through low cost methods. For example, the management of talipes through casting is relatively inexpensive, is widely available, and with proper compliance will prevent disability.

Our estimates however have to be considered as best-available data, as there was high heterogeneity among the studies, also reported in previous meta-analysis on NTDs in India [72, 73]. Due to the time-period included in the analyses, the definitions of anomalies varied, although the system-wise categorization of major anomalies was not too deviant from the International Classification of Diseases Version 10 (ICD-10) classifications. Stratified analysis over time did not yield any meaningful trends. It is noteworthy that institutional deliveries were only 26% in 1992–93 [74], but progressed to 79% in 2015–16 [75]. Birth defects data from studies conducted during the earlier period could be influenced by the high number of home births, and this could also be a limitation in the estimates. Most of the studies were hospital based. Community based studies were few, and none of the studies mentioned data on home births. For hospital based studies, the catchment areas of hospitals are undetermined due to high patient mobility. Furthermore, the studies included data from large public hospitals which frequently serve as referral centers for high risk mothers and complicated cases. Such methodological issues could be one of the reasons for the different rates observed for anencephaly versus spina bifida, as the latter is the more common condition [68]. Another factor influencing the estimates was that majority of the studies used only clinical assessment for case ascertainment. Incomplete ascertainment may therefore contribute to under-estimation of some anomalies. For example, the low prevalence of congenital heart defects as compared to available registry data could be ascribed to use of only physical examination at the time of birth [68]. Similarly, Down syndrome which is one of the most common birth defects, was not reported in most of the included studies. This discrepancy could be because our meta-analysis included studies that reported birth defects detected in the first week of life, while Down syndrome may be diagnosed after discharge. Another very important source of under-estimation would be the lack of data on termination of pregnancies due to fetal anomaly, as none of the studies reported this data. It should also be pointed out here that only PubMed was used for search of articles, and there may be a possibility that some articles were missed.

Despite these limitations, this review is important, as it is the first to report the magnitude of birth defects in India, and the need to establish a systematic method of surveillance for these conditions. The first point arising from the study is to determine whether surveillance for birth defects in India should be hospital or population based [12]. Data from a network of hospitals forms the cornerstone of existing birth defects registries in developed nations. Apart from systematic data collection, all deliveries occur at hospitals in these settings, and the populations accessing these hospitals are more or less well defined. In contrast, hospital based data will be inappropriate for India due to a number of reasons. Firstly, presence of large numbers of private hospitals would make inclusion of all in the reporting network difficult, leading to a risk of under-estimation of the number of cases. Till date, data reporting is not mandatory from private hospitals in India. Inappropriate inclusion of a major referral hospital, or a hospital providing free services into a birth defects surveillance network could also mislead estimates. Furthermore, unlike the well demarcated populations catered to by hospitals in developed countries, the population catered to by hospitals in India are extremely heterogeneous. There is significant patient mobility, as most healthcare is choice based, and financed through personal expenditure. The hospitals could cater to maternity cases from any part of the country, and these could include mothers from rural or urban areas. Cultural practices, such as preferred delivery at maternal residence could also confound results on geographic distribution of birth defects. All these factors highlight that hospital based surveillance could yield poor quality data, and may even misguide health services by suggesting occurrence of clustering of cases due to improper selection of hospitals into the reporting network.

Under such circumstances, true data on birth defects can be obtained from population-based birth defects surveillance, with active surveillance from carefully selected populations, identified in different parts of the country. One of the major functions of birth defects registries is to monitor maternal exposures, such as the prevalence of micronutrient deficiencies, poor maternal health status, agricultural lifestyles or other occupational exposures. India has several high risk situations and areas. Industrial catastrophes like the Bhopal gas tragedy or reports of children with severe birth defects in areas where banned pesticides are being used are examples of populations where communities can be selected, and long term surveillance can be initiated. Many of the risk exposures are shared for several other adverse pregnancy outcomes. Thus, surveillance for birth defects will be an added asset to monitoring maternal and child health outcomes in the country. In addition to selection of population based sites for surveillance, there is the need for adopting standardized definitions and methodology for case ascertainment. Inclusion of data on elective terminations of pregnancy after detection of fetal anomalies and follow-up of infants to include anomalies detected at later ages are important considerations when planning surveillance to reduce under estimation. For countries with limited resources the recently published manual for birth defects surveillance is an excellent tool that enlists steps for facilitating birth defects surveillance [12]. Use of such tools and population based surveillance will permit comparison of data on birth defects in low income countries with those reported from existing congenital anomaly registries.

## Conclusions

In conclusion, this meta-analysis identified that as many as 472,177 (421,652 to 522,676) births affected by congenital anomalies may be occurring in India each year, with anencephaly and talipes being the most frequently reported anomalies. The high reporting of anencephaly suggests the need for a preconception folic acid supplementation programme, but nation-wide studies on implementation have to be conducted. The occurrence of talipes and other anomalies requiring surgical correction suggests the need to strengthen referral services for treatment/management of children born with birth defects. In terms of public health implications, the meta-analysis was unable to identify data on the impact of congenital anomalies on neonatal mortality. The impact of congenital anomalies on childhood disability was however apparent as both anencephaly and talipes are potentially disabling conditions. The analysis identified the need for future studies using standard definitions and methodology so that the data would be globally comparable. In terms of hospital versus population based surveillance, the analysis suggested the need for establishing population based registries with active surveillance for birth defects and maternal risk exposures from carefully selected populations.

## Abbreviations

CI: Confidence Interval
*NM*: Not mentioned
NTDs: Neural tube defects
